# Central Diabetes Insipidus and Hyperglycemic Hyperosmolar State Following Accidental Carbon Monoxide Poisoning

**DOI:** 10.7759/cureus.1305

**Published:** 2017-06-03

**Authors:** Zain Ul Abideen, Syed Nayer Mahmud, Amna Rasheed, Yusaf Farooq Qasim, Furqan Ali

**Affiliations:** 1 Nephrology and Renal Transplant, Shifa International Hospital, Islamabad, Pakistan; 2 Department of Internal Medicine, Shifa International Hospital, Islamabad, Pakistan; 3 Medicine, Shifa International Hospital, Islamabad, Pakistan; 4 Nephrology, Shifa International Hospital, Islamabad, Pakistan

**Keywords:** carbon monoxide, hyperosmolar hyperglycemic state, central diabetes insipidus, hypernatremia, polyuria

## Abstract

Carbon monoxide poisoning is common and carries significant morbidity and mortality. The nervous system, particularly the brain, is frequently affected by it, owing to its high metabolic activity and oxygen requirements. Carbon monoxide damages the nervous system by both hypoxic and inflammatory mechanisms. Central diabetes insipidus is an extremely rare complication of carbon monoxide poisoning. Herein, we report the case of a young lady, who developed this complication and severe hypernatremia after accidental carbon monoxide poisoning. She also developed a hyperglycemic hyperosmolar state during the treatment for hypernatremia. To the best of our knowledge, both these entities have not been reported together in association with carbon monoxide poisoning. The purpose of this article is to emphasize the anticipation and early recognition of central diabetes insipidus in carbon monoxide poisoning. This can prevent severe hypernatremia and complications associated with its presence and treatment.

## Introduction

Carbon monoxide (CO) is a common cause of death in various parts of the world and carries considerable morbidity and mortality [1-2]. CO poisoning can result in both short and long term complications and can affect any organ of the human body. The effects on the heart and brain are particularly profound because of their high metabolic activity and oxygen requirements [2]. A broad spectrum of short and long-term neurological complications can occur, and this is postulated to be secondary to both hypoxic and inflammatory injuries to the brain parenchyma [3]. Central diabetes insipidus (CDI) is a rare occurrence after CO poisoning with only a few cases reported in medical literature. The exact mechanism of its occurrence is also unknown. We present the intriguing case of a young lady, admitted to our hospital with accidental CO poisoning. She developed polyuria, severe hypernatremia, and hypovolemic shock. A very high serum osmolarity, low urine osmolarity and response to desmopressin were suggestive of CDI as the culprit disorder. Our patient also developed a hyperglycemic hyperosmolar state (HHS). We believe this was probably secondary to the profound circulatory stress, volume depletion, and iatrogenic causes (Intravenous steroids and dextrose infusion). Despite correction of this state with insulin, polyuria did not improve, for which a diagnosis of CDI was considered.

The purpose of reporting this case is to highlight the importance of anticipation and early recognition of CDI in CO poisoning with an aim to intervene as soon as possible and avoid neurological damage and cerebral edema. In addition, these patients are at risk for developing severe hyperglycemia due to stress, dehydration, and iatrogenic causes. HHS and CDI have been reported simultaneously in patients with hypoxic-ischemic encephalopathy (HIE) post cardiac arrest, however, to the best of our knowledge the coexistence of these two conditions after CO poisoning has not been reported.

## Case presentation

A 24-year-old lady, a resident of Islamabad, was received in the emergency department after she was found unconscious in the washroom. She had been in the washroom for 30 minutes. A gas water geyser was fitted inside the washroom. She was initially rushed to a local hospital where she was treated with high flow oxygen and was referred to our hospital within two hours. There was no other medical or surgical history of note. She was a non-smoker and had no history of drug abuse. On examination, she had a depressed conscious level; her Glasgow Coma Scale (GCS) score was four (E1, V1, M2). Her pupils were reactive to light and plantar response was flexor in both feet. Her pulse rate was 98/min, blood pressure 110/80 mm Hg, respiratory rate 17/min, oxygen saturation 88% on room air and she was afebrile. There was no focal neurological sign or evidence of any head injury. Examination of the cardiovascular, respiratory, and gastrointestinal systems was unremarkable. She was intubated and ventilated as soon as she arrived in the emergency department.

The arterial blood gas revealed blood pH of 7.31, pCO2 32.9 mm Hg, PaO2 81.3 mm Hg and bicarbonate 16.5 mEq/l, on a fraction of inspired oxygen (FiO2) of a hundred percent. Her carboxyhemoglobin levels were 22.5% checked approximately two hours after she was found unconscious.

Important lab parameters on admission are tabulated in Table 1.

**Table 1:** Laboratory parameters on admission

Parameter	Patient value	Reference range
Hemoglobin	13.3 g/dL	11.5-16.5 g/dL for females
White cell count	16,000/cumm	4-11,000/cumm
Platelet count	239,000	150,000-450,000/cumm
Serum creatinine	1.28 mg/dL	Less than 1.2 mg/dL
Serum sodium	139 meq/L	135-145 meq/L
Serum potassium	5.2 meq/L	3.5-5.0 meq/L
Serum urea	29.9 mg/dL	Less than 20 mg/dL
Blood sugar random	345 mg/dL	<200 mg/dL

Urine examination was completely normal. Urine drug toxicology screen was negative and liver function tests were within normal limits. Her electrocardiogram (ECG) was consistent with sinus tachycardia and initial computed tomogram (CT) scan of the brain and chest X-ray were unremarkable. She was transferred to the medical intensive care unit (ICU) for mechanical ventilation on volume controlled continuous mechanical ventilation with FiO2 of a hundred percent and positive end-expiratory pressure (PEEP) 5 cm of H2O. She was given an intravenous infusion of isotonic saline, intravenous antibiotics and deep vein thrombosis prophylaxis with enoxaparin.

On the second day of admission, her GCS was static. Her blood pressure was 140/80 mm Hg and heart rate 93/minute. Her serum sodium increased to 161 mEq/L. The intravenous fluids were changed first to half saline and then to 5% Dextrose water at 100 ml/hour. Samples repeated in the day revealed persistently rising sodium levels and the one done in the night showed serum sodium of 205 meq/L at which a Nephrology consult was called. The urine output was 6400 in the past 24 hours. At this point, the patient had signs of volume depletion; she had a dry tongue and mucous membranes, heart rate of 110/minute and a blood pressure of 90/40 mm Hg. A one-liter bolus of isotonic saline was given until patient’s blood pressure stabilized, water deficit was calculated (11 liters) and volume replacement was begun, with an aim to correct serum sodium by no more than 10 meq/24 hours. For this purpose, 5% dextrose water and free water boluses were used. Intravenous hydrocortisone was also started. In the morning, the next day, a random blood sugar level of 1399 mg/dL was reported. This was managed with an insulin infusion. The blood sugar normalized on the same day, however, the polyuria persisted.

**Figure 1:**
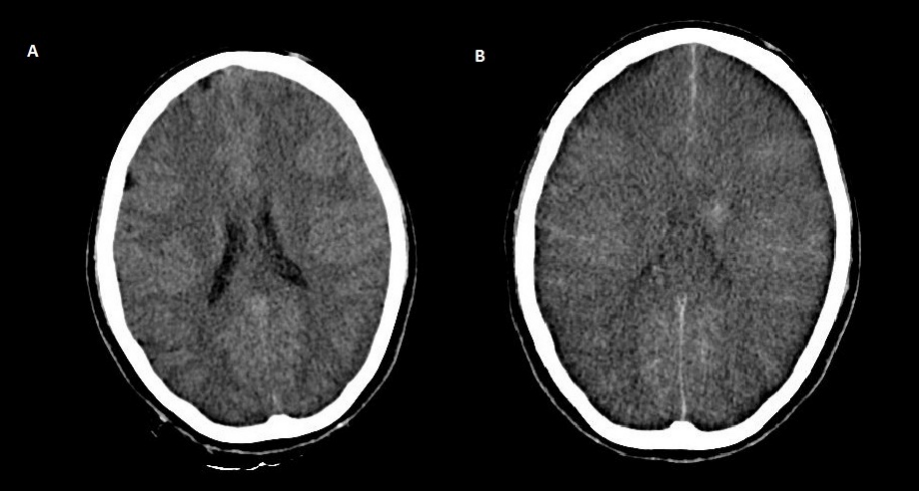
Computed tomography (CT) scans on admission and on the last day of admission Panel A - Normal CT scan on admission. No evidence of cerebral edema or infarction/bleeding. Panel B - Follow-up CT scan showing marked diffuse brain edema with effacement of internal and external cerebrospinal fluid (CSF) spaces suggestive of Global cerebral ischemia.

Labs sent at this stage revealed a urinary osmolality of 215 mOsm/kg and serum osmolality of 397 mOsm/kg. A diagnosis of central diabetes insipidus was suspected and tablet Desmopressin was started at 0.25 mg every 12 hours hourly via nasogastric tube. Post desmopressin, her urine osmolality increased to 378 mOsm/kg, and a day later to 480 mOsm/kg. The urine output also declined steadily to 2.3 liters/day. These observations were consistent with the diagnosis of central diabetes insipidus. Her serum sodium levels 24 hours later were 195 meq/L, falling to 184 meq/L, 173 meq/L and finally to 167 meq/L over the third, fourth and fifth day, respectively. The average rate of sodium reduction was 9.5 meq/day. The two-dimensional echocardiogram was essentially normal. A follow-up CT scan of brain on the fifth day revealed marked diffuse brain edema with effacement of internal and external cerebrospinal fluid (CSF) spaces suggestive of global cerebral ischemia. The normal CT scan on admission is shown in Figure 1A, while that done on the fifth day in Figure 1B. The electroencephalogram (EEG) findings were also consistent with a severe hypoxic-ischemic cerebral insult. She was referred to another hospital on the fifth day of her admission where she succumbed to her illness.

## Discussion

CO poisoning may be accidental or suicidal. The most common source of CO emission leading to accidental poisoning is inadequately ventilated gas appliances, fires, and exhaust fumes. Our case was accidental, caused by an inadequately ventilated bathroom water heater. CO poisoning inflicts a number of adverse outcomes on the nervous system. It causes severe hypoxic injury to the brain parenchyma. The areas of the brain most vulnerable include the cerebral cortex, white matter, and the basal ganglia (more commonly globus pallidus) [2-3]. Up to 30% patients with CO poisoning may go on to develop neurological sequel [4]. These may include short-term memory loss, psychosis, gait and speech disorders, Parkinsonism, cortical blindness, and depression. A recent study linked it to the development of dementia [1]. Despite all these disorders occurring quite frequently, CDI is an extremely rare complication of CO poisoning. We searched Pub Med and could only find three patient records. All of these cases were accidental. The rarity of this complication, despite CO poisoning being very common, may be because of the rich blood supply endowed to the posterior pituitary gland [5]. This might explain why CDI occasionally occurs after extensive hypoxic brain injury post cardiopulmonary arrest [6].

CDI results from a decrease or absence of the antidiuretic hormone (ADH) which is synthesized in the hypothalamus, transported to and released from the posterior pituitary gland. Mostly, the disorder is idiopathic. Important causes include trauma, pituitary surgery, hypoxic or ischemic encephalopathy and the disease may be familial [7]. The history, lab analysis, and clinical picture in our patient were consistent with carbon monoxide as a cause for CDI and other causes of CDI had been excluded. The most common presentation in CDI is with abrupt polyuria and our patient had a urine output in excess of six liters per day. Patients with CDI may suffer from moderate to severe hypernatremia if deprived of water [8]. Our patient had very severe hypernatremia as stated above.

CDI is diagnosed by means of the water deprivation test and response to desmopressin [7]. The former was technically not possible in our patient as she was on a ventilator. The very high serum sodium levels and low urine osmolality were indicative of diabetes insipidus (DI) as the culprit disorder. Our patient responded to desmopressin with an almost 50% reduction of her urine output, which supported the diagnosis of CDI; in addition, her urine osmolality increased and her serum osmolality and sodium levels declined with this treatment. The treatment of CDI involves decreasing the urine output by increasing the activity of ADH. In addition, replacement of ongoing volume losses and correction of hypernatremia is essential. Desmopressin (dDAVP) is the preferred treatment [7]. It can be given via nasal insufflations, subcutaneously, intravenously, or orally (tablets). We used the tablet formulation, as it was the only form available at our center at that time. We used a dosage of 0.2 mg twice daily, to which the patient responded well. The hypernatremia was also treated, by calculating the “water deficit” and replacing it to cautiously correct sodium levels to ≤10 meq/day. The fluid used was 5% dextrose in water, while initially one-liter bolus of normal saline was used when the patient was in shock.

Hypernatremia above 160 meq/L can lead to brain shrinkage, intracranial bleeding, and seizures. Our patient had her sodium levels reduced cautiously and despite that, developed new onset cerebral edema. Chang and Lin described a similar course of events; in the only available case records of CO-induced CDI, both patients developed cerebral edema while one also developed subarachnoid hemorrhage [9]. CO poisoning itself can cause cerebral edema, whereas the reduction in sodium levels may have worsened this. These observations suggest that serum sodium levels should be corrected even more gradually in patients with CO poisoning who are already at risk of cerebral edema.

HHS has been reported simultaneously with CDI in patients with severe hypoxic ischemic encephalopathy. The causes described usually include profound stress, steroids and intravenous dextrose infusions [10]. All these factors were present in our patient. Past cases of CDI secondary to CO poisoning, however, have not described HHS simultaneously. Ours is the first case, which reports the simultaneous occurrence of both these entities in a patient with accidental CO poisoning.

## Conclusions

Central diabetes insipidus and the hyperglycemic hyperosmolar state may occur simultaneously in severe carbon monoxide poisoning. The latter can occur during correction of severe hypernatremia with the dextrose solution. The occurrence of cerebral edema in this patient, despite the non-rapid reduction of hypernatremia, suggests that these patients are at a greater risk for cerebral edema. Early anticipation and recognition of CDI in CO poisoning is essential to prevent severe neurological and metabolic complications.
